# Stepwise large genome assembly approach: a case of Siberian larch (*Larix sibirica* Ledeb)

**DOI:** 10.1186/s12859-018-2570-y

**Published:** 2019-02-05

**Authors:** Dmitry A. Kuzmin, Sergey I. Feranchuk, Vadim V. Sharov, Alexander N. Cybin, Stepan V. Makolov, Yuliya A. Putintseva, Natalya V. Oreshkova, Konstantin V. Krutovsky

**Affiliations:** 1 Laboratory of Forest Genomics, Genome Research and Education Center, Siberian Federal University, 660036 Krasnoyarsk, Russia; 2 Department of High Performance Computing, Institute of Space and Information Technologies, Siberian Federal University, 660074 Krasnoyarsk, Russia; 3 Department of Informatics, National Research Technical University, 664074 Irkutsk, Russia; 4 Limnological Institute, Siberian Branch of Russian Academy of Sciences, 664033 Irkutsk, Russia; 5 Laboratory of Forest Genetics and Selection, V. N. Sukachev Institute of Forest, Siberian Branch of Russian Academy of Sciences, 660036 Krasnoyarsk, Russia; 6 Department of Forest Genetics and Forest Tree Breeding, Georg-August University of Göttingen, 37077 Göttingen, Germany; 7 Laboratory of Population Genetics, N. I. Vavilov Institute of General Genetics, Russian Academy of Sciences, Moscow, 119333 Russia; 8 Department of Ecosystem Science and Management, Texas A&M University, College Station, TX 77843-2138 USA

**Keywords:** de novo genome assembly, Siberian larch, *Larix sibirica*

## Abstract

**Background:**

De novo assembling of large genomes, such as in conifers (~ 12–30 Gbp), which also consist of ~ 80% of repetitive DNA, is a very complex and computationally intense endeavor. One of the main problems in assembling such genomes lays in computing limitations of nucleotide sequence assembly programs (DNA assemblers). As a rule, modern assemblers are usually designed to assemble genomes with a length not exceeding the length of the human genome (3.24 Gbp). Most assemblers cannot handle the amount of input sequence data required to provide sufficient coverage needed for a high-quality assembly.

**Results:**

An original stepwise method of de novo assembly by parts (sets), which allows to bypass the limitations of modern assemblers associated with a huge amount of data being processed, is presented in this paper. The results of numerical assembling experiments conducted using the model plant *Arabidopsis thaliana*, *Prunus persica* (peach) and four most popular assemblers, ABySS, SOAPdenovo, SPAdes, and CLC Assembly Cell, showed the validity and effectiveness of the proposed stepwise assembling method.

**Conclusion:**

Using the new stepwise de novo assembling method presented in the paper, the genome of Siberian larch, *Larix sibirica* Ledeb. (12.34 Gbp) was completely assembled de novo by the CLC Assembly Cell assembler. It is the first genome assembly for larch species in addition to only five other conifer genomes sequenced and assembled for *Picea abies*, *Picea glauca*, *Pinus taeda*, *Pinus lambertiana,* and *Pseudotsuga menziesii var. menziesii*.

**Electronic supplementary material:**

The online version of this article (10.1186/s12859-018-2570-y) contains supplementary material, which is available to authorized users.

## Background

The de novo assembling of large genomes, such as in conifers, that have the length of 12 to 30 Gbp and consist of about 80% of highly repetitive elements (repeats), is a rather complex task [1–12]. The main problem of assembling such genomes is the limitations of assembler programs. As a rule, modern assemblers are designed to assemble genomes shorter or equal to the length of the human genome (3 Gbp). Most assemblers cannot handle the amount of input sequence data required to provide the coverage needed for a high-quality assembly or take too much time and computer resources. This prompts the development of new approaches in assembling large genomes, including Siberian larch (*Larix sibirica* Ledeb.), which together with Siberian stone pine (*Pinus sibirica* Du Tour) are the main objects of the genome project “Genomics of the key boreal forest conifer species and their major phytopathogens in the Russian Federation” funded by the research grant No. 14.Y26.31.0004 from the Government of the Russian Federation.

## Methods

### A stepwise approach to assembling large genomes

High sequence coverage is always needed for high-quality de novo genome sequencing and assembly. For a given average genome coverage, the coverage of individual genome regions is approximately described by the Poisson distribution according to the Lander-Waterman theory [13]. Insufficient coverage increases the probability of zero coverage of some genome regions. Meanwhile, even a single coverage of genome regions is sufficient for their assembling using De Bruijn graph based methods [14] assuming no errors and repeats.

To solve the problem, a new stepwise approach to assembling large genomes “in parts” was developed. The idea of partitioning data to perform assembly is not new. For example, in the article [15] it was proposed to apply a similar two-step hierarchical approach with the aim of improving the quality of assembly of bacterial genomes with very high coverage. However, the approach presented in [15] does not solve the problems of assembling large and super-large genomes, especially if DNA was obtained from diploid tissue.

In our case the assembly is also done in two steps. In the first step, the entire input pool of the sequence reads is divided into several sets (parts). The size of each set is within the  limit for the number of reads that can be handled by the assembler program. Each set is assembled separately, then the contigs obtained for each part are combined and used as the input data for the second step of assembling.

With this approach, the genome coverage by the input contigs no longer obeys the Poisson distribution in the second step of assembling. However, the level of coverage will not be greater than the number of parts by which the original pool of reads has been partitioned, which allows to bypass the limitation for the maximum amount of input data in the second step.

The challenge of the approach is the lower tolerance to sequencing errors and polymorphisms. The ambiguity in the input sequences in the second step could lead to generating duplications in the output. Therefore, the pipeline for the assembly with this approach should also include verification of the assembly for redundancy to exclude potential duplicates. We used the UCLUST package [16] and self-blasting for this task.

**Fig. 1:**
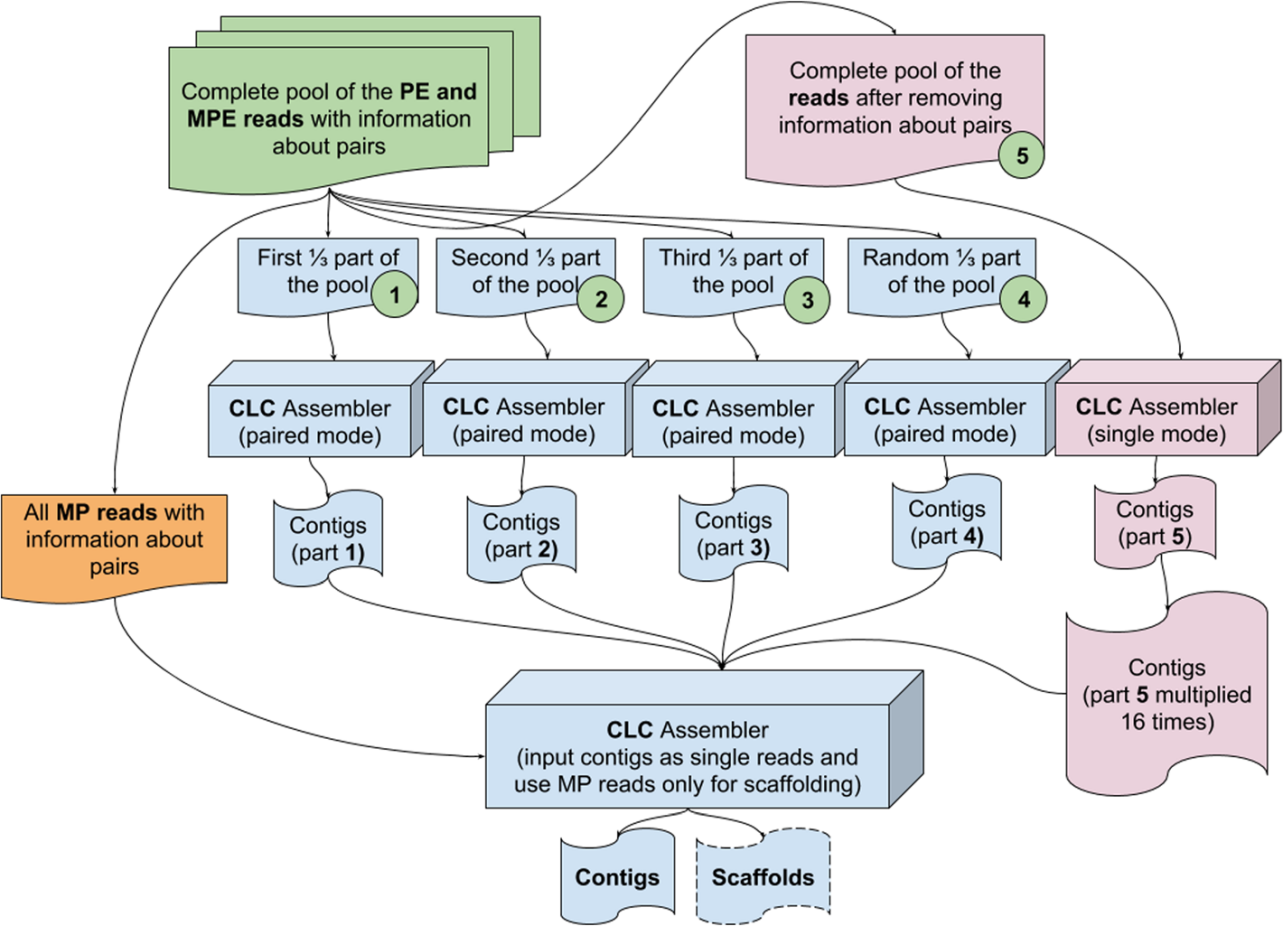
Stepwise assembly workflow chart

It should be noted that not all assembly programs allow generating contigs with a coverage below the threshold value. To overcome this obstacle in the second step of the stepwise assembly, either the program codes should be changed or the software that does not have these limitations, such as the CLC Assembly Cell (QIAGEN, Hilden, Germany), should be used. This software takes into account possible sequencing errors during assembling. Thus, if there are sequencing errors in the input reads, most of them will not be incorporated in the contigs generated in the first step for each part of the pool. However, the problem of the stepwise assembling could be insufficient coverage for each part, which can lead to shorter contigs. Since there is a restriction on the minimum length of contigs in the assembling programs, such short contigs with insufficient lengths will be excluded from the assembly. Therefore, to reduce the probability of gaps due to excluding short contigs in the second step, one of the sets in the first step included all reads from the original data pool, but to make computing possible, they were used as single end reads, and they were also multiplied. All steps are presented as a workflow chart in Fig. 1.

### Testing of the proposed stepwise approach on the model plant species *Arabidopsis thaliana*

To test the applicability of the proposed method of stepwise assembling for de novo assembling of large genomes, such as in *L. sibirica* (12.03 Gbp), a genome assembly of the model plant species *Arabidopsis thaliana* obtained by the proposed method was compared with the standard de novo assembly of this species genome. A relatively small subset of *A. thaliana* genomic reads was selected to get a genome coverage comparable to *L. sibirica.*

As an additional argument supporting the applicability of the method, the histograms of genome coverage obtained for *A. thaliana* and *L. sibirica* were compared for similarity. To construct the histograms, the genomic reads used for assembling were mapped to the assembled genomes using the bowtie software [17] for *A. thaliana* and the CLC read mapper for *L. sibirica*.

**Fig. 2:**
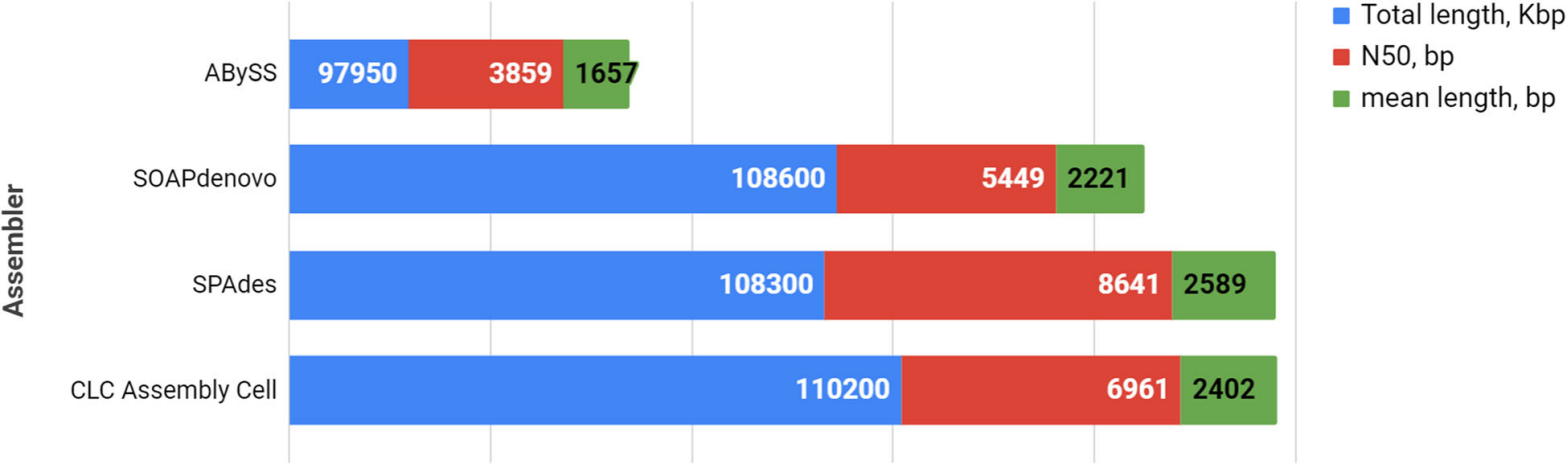
The results of the traditional de novo *Arabidopsis thaliana* genome assembly generated using four different assemblers. Minimum contig length used for assembling was 200 bp

The *A. thaliana* genome contains 5 chromosomes and 135 Mbp [18]. We used the SPAdes [19], AbySS [20], CLC Assembly Cell (https://www.qiagenbioinformatics.com/products/clc-assembly-cell), and SOAPdenovo [21] assemblers for the traditional de novo assembly of the *A. thaliana* genome. The genomic paired-end reads of *A. thaliana* were downloaded from the Genbank SRA database (accession number SRR492411 [22]). The results of assembly at the level of contigs by different assemblers are presented in Fig. 2 and Additional file 1: Table S1.

**Fig. 3:**
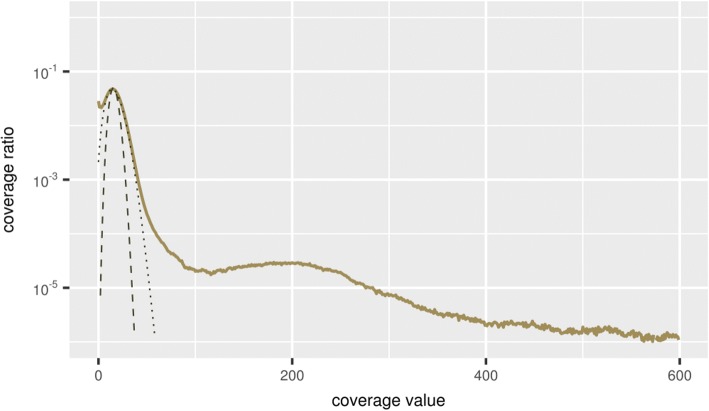
Histogram of the *Arabidopsis thaliana* genome coverage by the mapped reads used for the genome assembly generated by the CLC Assembly Cell software (solid line). Expected and corrected Poisson distributions are represented by dashed and dotted lines, respectively. The number of reads (degree of the genome coverage) is on the horizontal axis; the logarithmic proportion of the genome with such degree of coverage is on the vertical axis

The result of assembling repetitive regions of the genome depends on the number and similarity of copies of a particular type of repeat. With a small and divergent number of copies, the assembler program, as a rule, is able to separate individual copies, so that all variants of this repeat will be presented in the final contigs. With a large number of identical or nearly identical copies of the same type, it would be difficult for an assembler to separate them. The number of repeats in the genome of *A. thaliana* represents quite a significant part, according to different estimates, from 23 to 32% [23, 24]. As a result, in the final assemblies, identical repeats of the same type can be represented by a single contig. This was reflected in the histogram of the contig coverage based on the distribution of mapped reads used for assembling and presented in Fig. 3.

It should also be noted that in the area of maximum coverage its distribution is more accurately described by the corrected Poisson distribution expressed by the formula $\frac{bL^{bx}e\left(- bL\right)}{\Gamma \left( bx+1\right)}$, where *L* - average coverage, *x* - coverage value, *b* - correction parameter (inversed value of extended variation) (Fig. 3, dotted line, *b* = 0.3).

The observed coverage histogram followed the Lander-Waterman theory in general, and the degree of coverage can be approximately described by the Poisson distribution for the most of the genome with the left side maximum peak equalling 16 reads (Fig. 3). The exact fitting of the coverage histogram to the Poisson distribution and the corrected (over-dispersed) Poisson distribution were estimated using the iterative maximum likelihood-based procedure implemented in the R statistical package. The results of these tests confirmed the fitting of the histogram to the over-dispersed Poisson distribution around the peak value, with the reservations about semi-qualitative description of the distribution. The left and right tails of the distribution do not obey the provided model and should be described using other approaches. Because of this, the goodness of the fitting depends on the selection of limits around the peak value of distribution. In reasonable limits between 0.5X and 2X of peak value, the match to over-dispersed Poisson distribution was significant based on the Kolmogorov-Smirnov (KS) test (*P* < 0.01), but the estimated values of parameters should be anyway considered as approximate to avoid an excess of accuracy.

The clearly observed “heavy tail” in the right part of the distribution for contigs with high coverage (more than 100 reads) could be explained by the highly repetitive elements that represented different parts in the original genome, but were aligned and mapped together to the same single contigs. Therefore, the observed coverage histogram can be divided into two parts, with a coverage of less or more than 100 reads, respectively. The key observation was that the observed coverage histogram for the *L. sibirica* genome followed the same trend that further confirms the applicability of the proposed method (respective larch data and figures are presented and discussed below in Results). The “heavy tails” were also observed in the coverage histograms in metagenomics [25] and medical DNA sequencing [26].

The number of copies of different types of repeats in the genome is governed by different evolutionary factors, and the simplest way to explain the heavy tail of the distribution is to use the Zipf’s law to describe the frequencies of different types of repeats [27]. According to the Zipf’s law, the frequencies of different types of repeats, sorted by the degree of occurrence, should be distributed in proportion to 1/*n*, where *n* is a consecutive number of the type of repeat in the list of observed types.

**Fig. 4:**
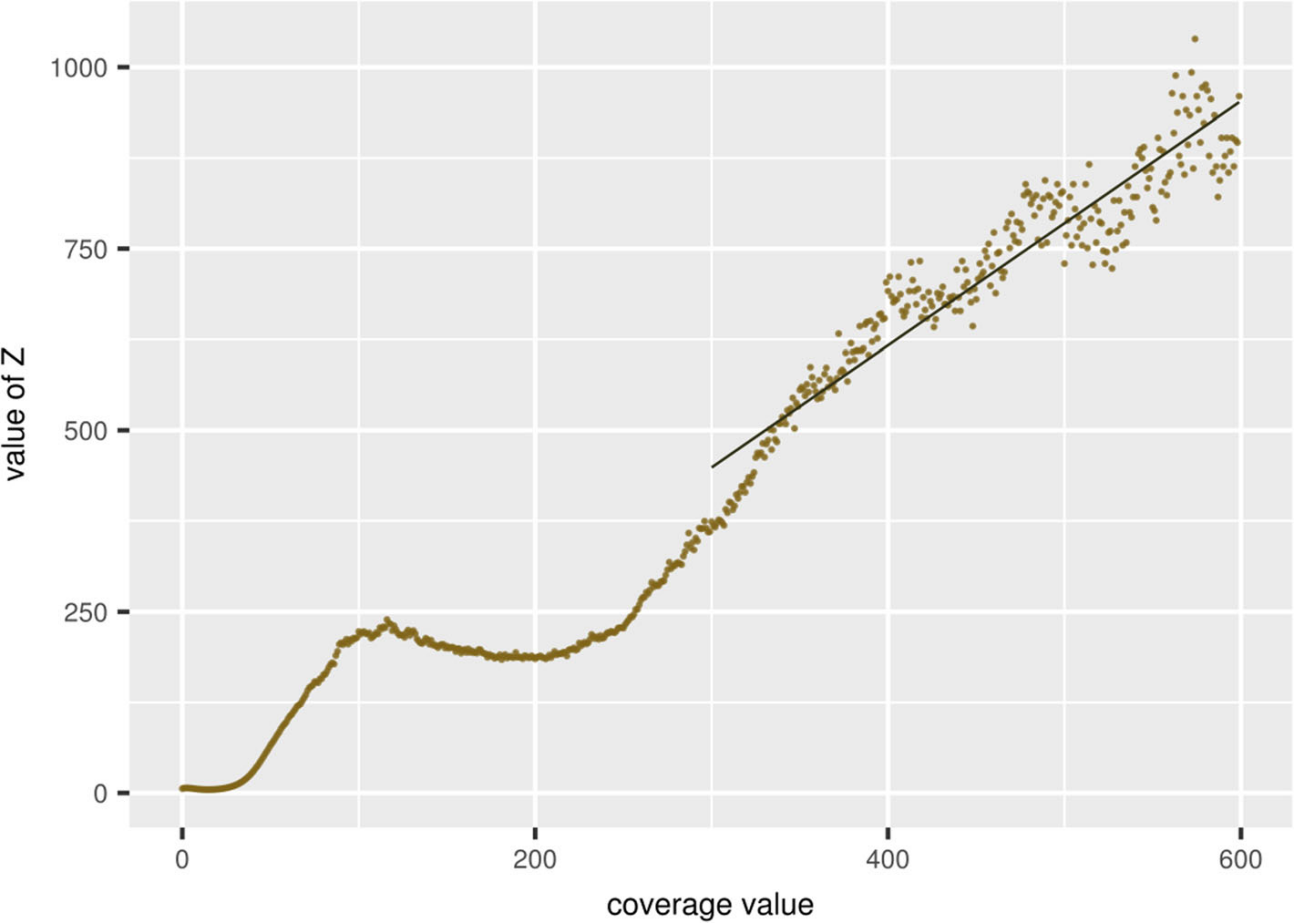
Dependence of the transformed value of the fraction of the genome coverage *Z* on the level of coverage. Solid line represents linear dependency calculated by the least square fit

The number of repeats with a given degree of coverage can be expressed as the derivative of this dependence, that is, in proportion to 1/*n*^2^, where *n* is the degree of coverage. If the value of $Z=\frac{1}{\sqrt{Y}}$ is calculated for a coverage histogram same as in Fig. 3, where *Y* is the percentage of the genome with a given degree of coverage, then according to the Zipf’s law, the value of *Z* should directly and proportionality depend on the degree of coverage. This dependence is demonstrated in Fig. 4 for the histogram of the observed coverage presented in Fig. 3.

As it can be seen from Fig. 4, the Zipf’s law is approximately satisfied for the coverage of more than 200 reads per site, which agrees with the abovementioned conclusion about the assembling repeats that occurred with different frequency in the genome. For a more accurate description of the observed dependence, it is recommended to use a distribution based on the Zipf-Mandelbrot law formulated as $\frac{1}{n^k}$, where *k* is generally different from unity [27]. Nevertheless, the applicability of this law to genomic nucleotide sequences requires further study.

There are a few studies of the *A. thaliana* genome that identified different types of repeats, using, in particular, the method of clustering repeat sequences (for example, [23, 24]). According to these studies, while there was a general tendency to meet the Zipf’s law for regions with a high degree coverage, individual peaks also appeared in the coverage distributions, such as in our case (Fig. 4), which can be interpreted as a manifestation of the similarity between individual types of repeats.

As shown in Fig. 3, the *A. thaliana* genome coverage was mostly described by the Poisson distribution with an average value of about 16 reads. To test the suggested stepwise assembling method, four sets were generated from the original pool of about 13 million reads. The first three sets included the first, second and third thirds of the original pool of reads, respectively. The fourth set also included one third of the original pool of reads, but was generated by random sampling from the mixed original pool of reads.

**Fig. 5:**
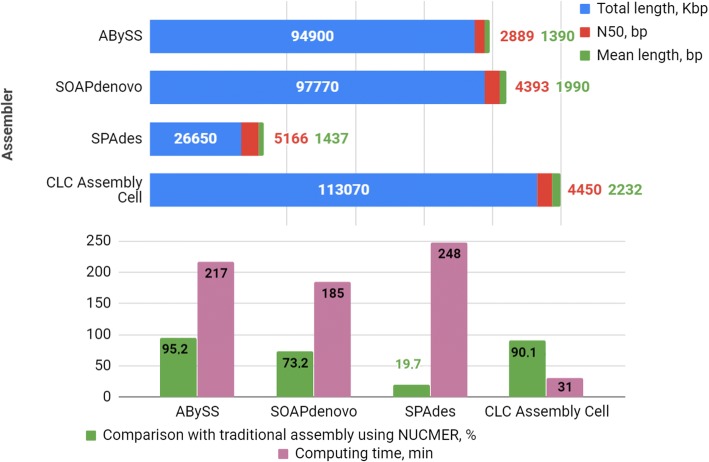
Results of the *Arabidopsis thaliana* genome stepwise assembly by different assemblers using raw reads partitioned into four sets. Minimum contig length used for assembling was 200 bp

Thus, four sets of reads were generated from the original pool of reads used in the tests presented in Fig. 2 and Additional file 1: Table S1. Figure 5 and Additional file 2: Table S2 presents the results of the stepwise assembly by four assemblers when each of the sets (parts) was assembled separately in the first step and then finally assembled by pooling all contigs from all four sets. It can be seen from the table that the CLC Assembly Cell demonstrated the best performance.

Table 1 presents the results of assembly of each of the sets (parts) separately (the first step), as well as based on the pooling of contigs obtained respectively from two, three, and four sets (parts) using the CLC Assembly Cell software.

**Table 1:** Results of the *Arabidopsis thaliana* genome stepwise assembling in four sets (parts) using the CLC Assembly Cell software

Assembly part	Total length, Mbp	Contigs		
		N50, bp	Number	Mean length, bp
1^a^	101.2	1586	110,067	919
2^a^	101.2	1601	109,903	920
3^a^	101.2	1595	110,119	919
4^b^	101.2	1586	110,384	916
1 + 2	113.2	3225	64,543	1753
1 + 2 + 3	116.6	3861	60,606	1923
1 + 2 + 3 + 4	113.7	4325	52,576	2161

^a^Represents approximately 1/3 of all original reads; ^b^Represents also approximately 1/3 of all original reads, but randomly selected

Table 1 shows that insufficient coverage led to a significant decrease in the average contig length compared to the data in Fig. 2 and Additional file 1: Table S1, but in the second step of assembling this parameter was corrected, and with the increase in the number of parts it was stabilized at the level of values close to the values obtained by the different assemblers used to assemble the entire pool of reads simultaneously.

The identity of assembly obtained using parts and the stepwise method with assembly based on assembling simultaneously all reads was tested by the NUCmer software (http://mummer.sourceforge.net), and the highest similarity was obtained for alignments generated by the CLC Assembly Cell (90.14%) and Abyss (95.24%) software, respectively (Fig. 5 and Additional file 2: Table S2), but the former software computed the assembly with a fewer number of contigs and a more realistic total length, and it computed it seven times faster than the latter one with the same computer hardware resources (31 vs. 217 min, Fig. 5 and Additional file 2: Table S2).

**Fig. 6:**
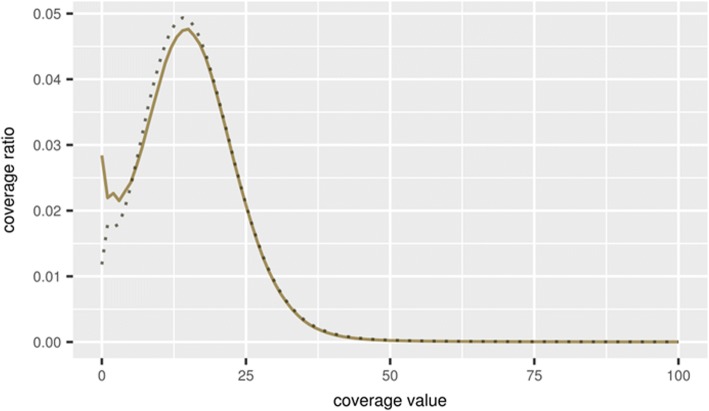
Comparison of the *Arabidopsis thaliana* genome coverage histograms obtained for the genome assembly assembled by the CLC Assembly Cell using all reads simultaneously (solid line) and the stepwise method with two steps and four parts (dotted line)

Figure 6 compares the genome coverage histograms for the *A. thaliana* genome assembly based on assembling the entire pool of reads simultaneously, such as in Fig. 3, and the assembly based on the stepwise assembling in two steps of four parts (Table 1). It is clearly seen in Fig. 6 that the stepwise assembled genome was adequately covered by the original set of reads.

The ambiguous positions in the *A. thaliana* sequencing data were estimated by aligning original *A. thaliana* reads to the assembly by Bowtie2. They represented 0.7% of genome size. The duplications of contigs were not detected in the final assembly, thus indicating a low level of ambiguity for the assembly obtained by the suggested method.

### The stepwise approach for the *Larix sibirica* genome assembly

**Fig. 7:**
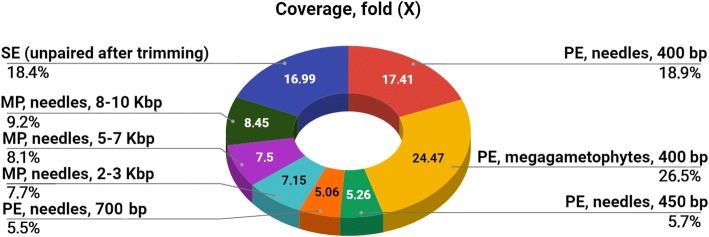
Sequence coverage for seven sequencing libraries used for the *Larix sibirica* genome assembly

For the assembly of the *L. sibirica* genome, four PE and three MP libraries with different insert size were used (Fig. 7 and Additional file 3: Table S3). In the first step, MPE libraries were decoupled and used as single reads to complete a pool of reads. The pool of reads was split to four parts and four sets of contigs were obtained, respectively. The CLC Assembly Cell software was selected for assembling the larch genome as the best performing software.

Also, a fifth set of reads was added to the analysis. This set included all reads, but the PE and MPE reads were decoupled and used as single reads. This set was generated because we found experimentally that the CLC Assembly Cell assembler was able to process the entire volume of the *L. sibirica* sequence data, but only if the information about the length of the insertion was not indicated. In this case the “Optimization of the graph using paired reads” step is skipped. In this step long repeats are allowed, and scaffolding is not performed which turns out to be too much computationally intense and practically prohibitive for large volume data. Therefore, this set increased the representation of all reads, but they all could be used only as the single end reads at this step.

**Fig. 8:**
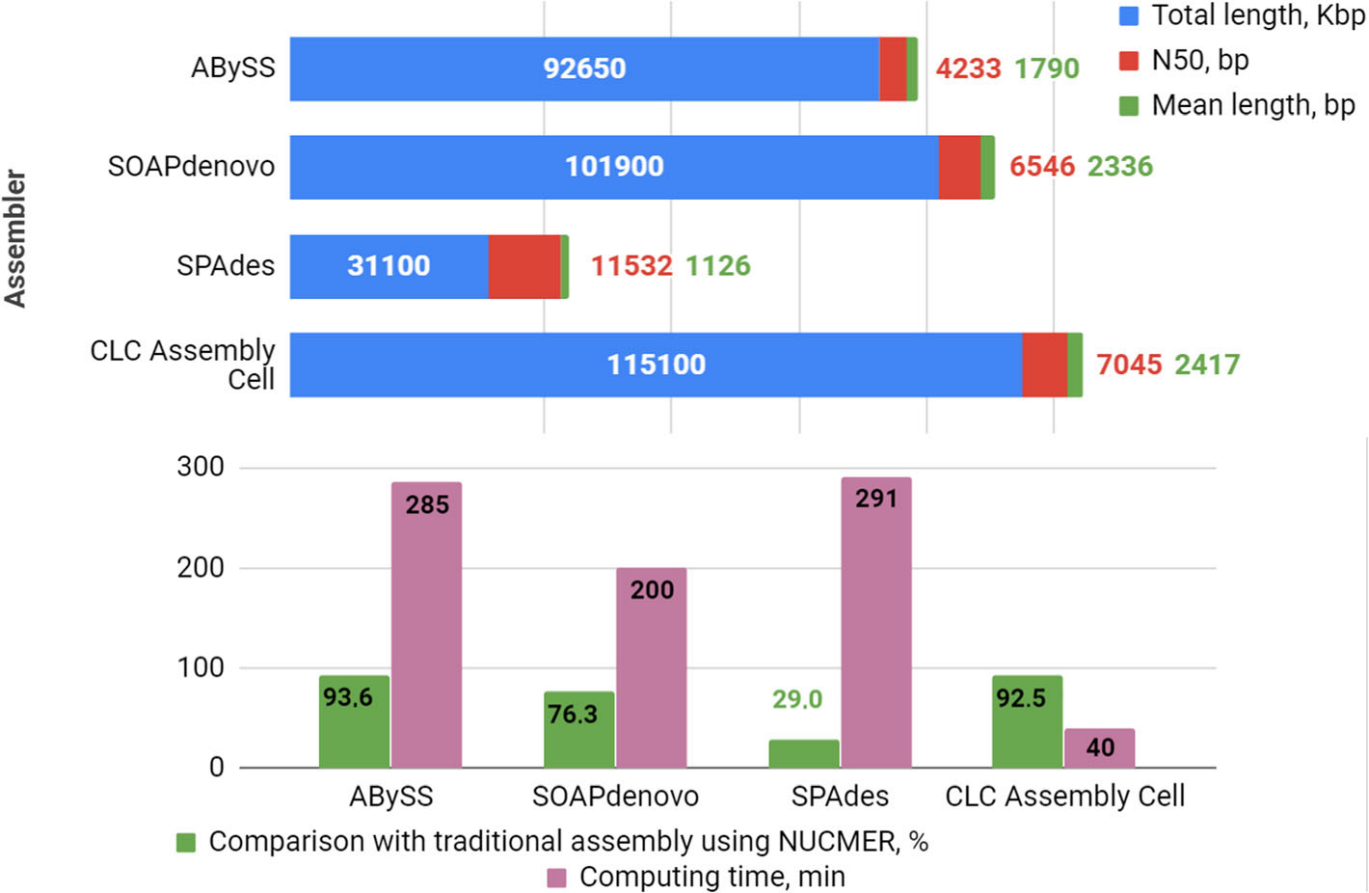
Results of the *Arabidopsis thaliana* genome stepwise assembly by four different assemblers using raw reads partitioned into five sets following the approach used for assembling of the *Larix sibirica* genome. Minimum contig length used for assembling was 200 bp

Unlike the inbred highly homozygous plant used for the genome sequencing and assembly, such as *A. thaliana*, the *L. sibirica* tree used for genome sequencing in our study represented a common forest tree with a relatively high level of individual heterozygosity and, respectively, high within individual biallelic variation. The number of ambiguous positions in the *L. sibirica* sequencing data was estimated at the level of 3.0% of the genome size. The presence of duplicate contigs was detected in the preliminary draft assembly of *L. sibirica* obtained in the second step, thus revealing the higher data ambiguity in the *L. sibirica* sequencing data compared to the *A. thaliana* data. To resolve the ambiguities in the second stage, the total number of all contigs resulting from the fifth set was increased by 16 folds by multiplying each contig 16 times, respectively. This trick allowed the CLC assembler to apply the majority rule when picking one of the alternative alleles, using the alleles selected in the fifths set in the first step of assembly. The same approach was used also for the *Arabidopsis thaliana* genome stepwise assembly by four different assemblers (Fig. 8 and Additional file 4: Table S4). The CLC Assembly Cell again demonstrated the best performance.

**Fig. 9:**
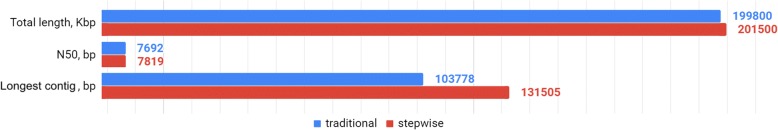
The traditional and stepwise CLC Assembly Cell genome assembly parameters for peach (*Prunus persica*). Minimum contig length used for assembling was 200 bp

In addition, to verify the accuracy of the stepwise CLC Assembly Cell assembly the medium size genome (265 Mb, 2n =16) of *Prunus persica* (peach) was also assembled by both the traditional method using 24,324,216 sequence reads (~15X coverage) available on https://www.ncbi.nlm.nih.gov/bioproject/PRJNA31227 and the same stepwise approach that was used for the larch genome assembly and based on the five parts (Fig. 9 and Additional file 5: Table S5). The traditional and stepwise assemblies were similar to 95.64% based on the NUCMER comparison.

## Results

### Stepwise assembly of the *Larix sibirica* genome in parts

The length of the *L. sibirica* genome is about 12.03 Gb [28], about 82% of which consists of repeats [6–8]. The volume of the larch sequencing data obtained (11 billion paired 100 bp long reads) was hardly manageable by the available genome assemblers and more than twice the maximum amount of data that the best performing software in our test with the *Arabidopsis* data CLC Assembly Cell can handle. Therefore, we developed a new stepwise assembly method for assembling this and other large genomes and demonstrated its consistency in computer experiments on assembling the model plant *A. thaliana* genome.

The original Siberian larch sequencing data were partitioned into five sets following mainly the procedure described for *A. thaliana* in Methods with an additional fifth set. Each set was separately assembled using the CLC Assembly Cell program. The assembly results are presented in Table 2 for each set. Only contigs with a minimum length of 200 bp were included in the final assembly.

**Table 2:** The assembly results of the five sets generated from the original *Larix sibirica* genome sequencing data

Set	Number of contigs	N50, bp	Maximum length, bp	Total length, Gbp
1	7,870,837	310	56,157	2.566
2	5,469,129	535	65,362	2.549
3	5,449,065	1383	157,662	4.319
4	4,677,717	1092	91,349	3.117
5	13,244,672	475	46,203	5.937

The total length of contigs assembled separately for each of the five sets varied from ~ 2.5 to ~ 6 Gb. The N50 parameter varied from ~ 300 to ~ 1300 bp. In the second step, individual assemblies were combined by specifying them as unpaired reads and changing the *k*-mer parameter length from 35 to 60. In addition, the mate pair (MP) reads generated from the MP libraries with 2000–10,000 bp long inserts were added to the CLC Assembly Cell input data. These reads were used at the stage of scaffolding (joining contigs into scaffolds with gaps of the known expected length).

Additional scaffolding was done using BESST [29], and 228,571 additional scaffolds were generated. The scaffolding was also improved by using larch transcriptome reads and RaScaf + Bowtie2 software [30]. About 92% of reads were mapped to the genome assembly and allowed us to connect 3622 contigs into scaffolds. The assembly was finished with gap-closing using the Sealer program implemented in the last part of the Abyss pipeline [31], and 61,037 gaps were closed.

Thus, the contigs of all five assemblies were processed and the obtained statistics is presented in Table 2.

Adding the last two assemblies based on the 4th and 5th sets (Table 2) improved the final parameters (Table 3) and increased the total contig and scaffold lengths from 7.18 to 7.99 Gb and from 11.04 to 12.34 Gb, respectively. The N50 parameter remained unchanged compared to the best values of partial assemblies. This is inconsistent with the results for *A. thaliana* assembly tests, but could be explained by the additional scaffolding procedure with the MP reads for the *L. sibirica* assembly.

**Table 3:** The final stepwise *Larix sibirica* genome assembly based on five sets and the MP reads

Assembly^a^	Number, mln	N50, bp	Maximum length, bp	Total length, Gbp
Contigs	12.40	1074	128,642	7.99
Scaffolds	11.33	6443	354,326	12.34

^a^Minimum contig length used for assembling was 200 bp

The assembly was tested for redundancy using a custom pipeline specially developed for this task, which checks for duplication taking into account possible erroneous nucleotide substitutions and indels. As a result, 74,851 scaffolds were excluded. The assembly was additionally checked for vector contamination and redundancy using the UniVec database (https://www.ncbi.nlm.nih.gov/tools/vecscreen/univec) and the BLAST program, and as a result, 10,681 sequences were deleted.

**Fig. 10:**
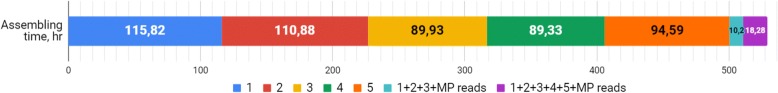
The computing time (number of hours) taken to assemble each set and the complete *Larix sibirica* genome using 40 cores

Finally, after scaffolding, a complete Siberian larch genome of 12.34 Gb was assembled de novo. The computing time taken to assemble the larch genome using 40 cores is presented in Fig. 10 and Additional file 6: Table S6. In total, it took about 529 h or 22 days. Therefore, the larch genome computing using the next best assembler SOAPdenovo could predictively take more than 100 days.

**Fig. 11:**
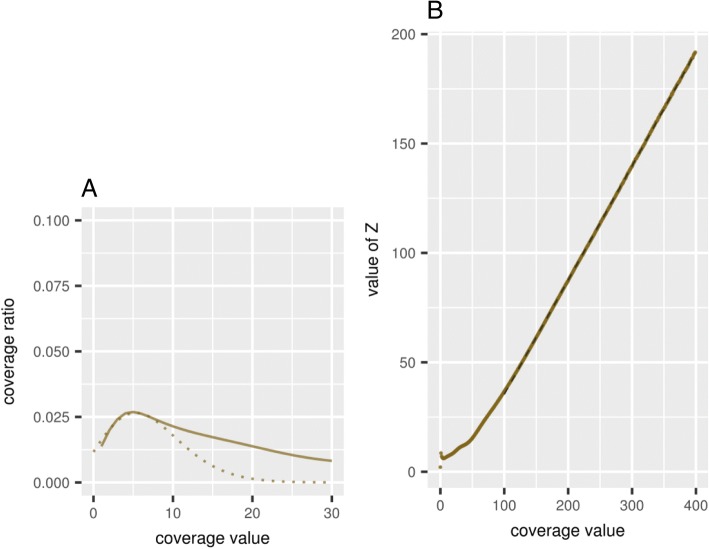
**a** The observed distribution of the Siberian larch genome coverage (solid line) and the expected one from the corrected Poisson distribution (dotted line) with the average coverage value equalling 7 and correction parameter *b* = 0.3. **b** Dependence of the transformed degree of genome coverage *Z* on the Siberian larch genome coverage (solid line). The dashed line represents linear dependency calculated by the least square fit and fully coincides with the solid line

The histogram of the coverage for the obtained genome corresponded to the Poisson distribution with extended variation in the regions with low coverage (Fig. 11a) and to the Zipf’s law in the region of high coverage (Fig. 11b) and was similar to the one obtained for *A. thaliana* (Fig. 3). The values for the inversed over-dispersion parameter were nearly the same for both genomes (0.3 ± 0.1), as it was confirmed by likelihood-based parameter estimates.

The correlation presented in Fig. 11b was completely linear in the region of sufficient coverage, as expected from the Zipf’s law, in contrast to the correlation for *A. thaliana*, in which many individual peaks were observed (Fig. 4). This is consistent with the results of the analysis of genomic repeats in Norway spruce [1], where it was difficult to cluster repeats and separate some types of repeats, as it can be done for many other genome sequences of eukaryotes. It also follows from our results that the distribution of repeats in conifers is continuous. The presence of a large number of repeats and discontinuities in assembling associated with them can explain the smaller average contig length in comparison with the results of the *A. thaliana* genome assembling.

**Fig. 12:**
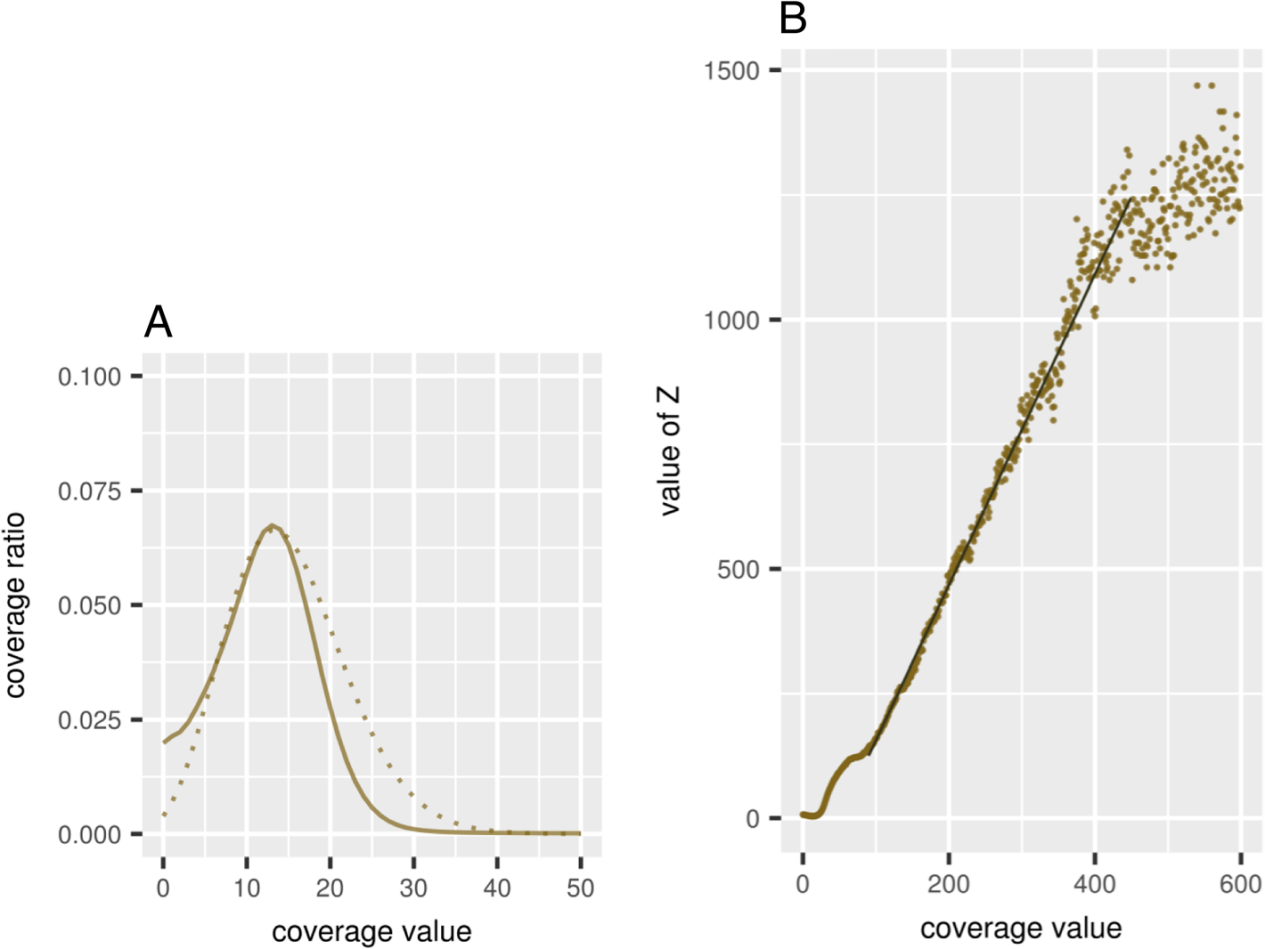
**a** The observed distribution of *Prunus persica* (peach) genome coverage (solid line) and the expected one from the corrected Poisson distribution (dotted line) with the average coverage value equalling 15 and correction parameter *b* = 0.3. **b** Dependence of the transformed degree of genome coverage *Z* on the peach genome coverage (solid line). The dashed line represents linear dependency calculated by the least square fit and fully coincides with the solid line

The accuracy of the stepwise CLC Assembly Cell assembly was also verified by assembling the medium size genome (265 Mb, 2n=16) of *Prunus persica* (peach) using both methods. The assembly parameters are presented in Table 3 and the histogram of the coverage - in Fig. 12. Both the observed and the expected distributions of the peach genome coverage were similar to those for *Arabidopsis* (Figs. 2, 3 and 5) and Siberian larch (Fig. 11) genomes.

The negative binomial distribution or the over-dispersed Poisson distribution is often used to describe genome coverage histograms, but, to our best knowledge, the effect of overdispersion was not systematically studied in the context of genome assemblies (but see [25, 32]). However, the similar values of the over-dispersion parameter for  the three assembled genomes confirmed by the KS tests could serve as an additional argument that the proposed method could be adequately scaled to the assembly of large genomes.

## Discussion

The testing of the proposed stepwise approach for assembling genomes in parts on the model plant species *A. thaliana* showed that, despite some deterioration of the distribution parameters of the contig lengths in the final assembly compared to normal assembling using the CLC Assembly Cell, the result of the stepwise assembling was comparable with the results of assembling all data simultaneously using different assemblers. The comparison of the lengths of the obtained genomes and histograms of the coverage obtained by different methods also allows us to state that the stepwise assembling by parts generates a consistent and reliable genome assembly corresponding to the original biological material.

The analysis of the coverage histograms carried out for *A. thaliana*, *Prunus persica* (peach) and larch showed a tendency to satisfy the Zipf’s law for the frequency of repeats and provided additional grounds for concluding that the stepwise assembly approach by parts is applicable for assembling large genomes, such as the Siberian larch genome. The interpretation of the coverage histograms using the Zipf’s law made it also possible to clarify the idea of statistical regularities characterizing the evolutionary mechanisms of multiplication of repeats in different plant species.

## Conclusion

Using the new stepwise de novo assembling method presented in the paper, the genome of Siberian larch, *Larix sibirica* Ledeb. (12.34 Gbp) was for the first time completely assembled de novo by the CLC Assembly Cell assembler. It is the first genome assembly for any larch species in addition to only five other conifer genomes sequenced and assembled for *Picea abies* [1], *Picea glauca* [2], *Pinus taeda* [3–5, 9, 11], *Pinus lambertiana* [10], and *Pseudotsuga menziesii var. menziesii* [12]. The presented approach makes assembling feasible for very large genomes with a reasonable computing time and without engaging huge computing resources. The assemblies produced using this approach are still of reasonable quality allowing their annotation and further use.

## Additional files

Additional file 1: **Table S1.** The results of the traditional de novo *Arabidopsis thaliana* genome assembly generated by four different assemblers. (DOCX 13 kb)

Additional file 2: **Table S2.** Results of the *Arabidopsis thaliana* genome stepwise assembly by different assemblers using raw reads partitioned into four sets. (DOCX 13 kb)

Additional file 3: **Table S3.** Sequencing libraries and generated sequence data used for the *Larix sibirica* genome assembly. (DOCX 14 kb)

Additional file 4: **Table S4.** Results of the *Arabidopsis thaliana* genome stepwise assembly by four different assemblers using raw reads partitioned into five sets following approach used for assembling of the *Larix sibirica* genome. (DOCX 14 kb)

Additional file 5: **Table S5.** The traditional and stepwise CLC Assembly Cell genome assembly parameters for peach (*Prunus persica*). (DOCX 13 kb)

Additional file 6: **Table S6.** The computing time taken to assemble each set and the complete *Larix sibirica* genome using 40 cores. (DOCX 13 kb)

## Abbreviations

bp: Base pair
Gb: Giga base
HPC: High performance computing
