# Supplementary material for: Stepwise large genome assembly approach: a case of Siberian larch (Larix sibirica Ledeb)
Source: BMC Bioinformatics. 2019 Feb 5;20(Suppl 1):37. doi: 10.1186/s12859-018-2570-y (PMC6362582; doi:10.1186/s12859-018-2570-y)
Supplement: Supplementary file 1 — Table S1. The results of the traditional de novo Arabidopsis thaliana genome assembly generated by four different assemblers. (DOCX 13 kb) [file 12859_2018_2570_MOESM1_ESM.docx]

**Additional file 1**

**Table S1**The results of the traditional *de novo* *Arabidopsis thaliana* genome assembly generated by four different assemblers*

| Assembler | Assembly | Total length, Mbp | N50, bp | number | mean length, bp |
| --- | --- | --- | --- | --- | --- |
| ABySS | contigs | 97.95 | 3811 | 59243 | 1653 |
|  | scaffolds | 97.95 | 3859 | 59083 | 1657 |
| SOAPdenovo | contigs | 104.8 | 3528 | 68318 | 1534 |
|  | scaffolds | 108.6 | 5449 | 48882 | 2221 |
| SPAdes | contigs | 108.2 | 8379 | 42217 | 2563 |
|  | scaffolds | 108.3 | 8641 | 41802 | 2589 |
| CLC Assembly Cell | contigs | 109.9 | 6653 | 44373 | 2232 |
|  | scaffolds | 110.2 | 6961 | 42328 | 2402 |

*Minimum contig length used for assembling was 200 bp.
