# Supplementary material for: Stepwise large genome assembly approach: a case of Siberian larch (Larix sibirica Ledeb)
Source: BMC Bioinformatics. 2019 Feb 5;20(Suppl 1):37. doi: 10.1186/s12859-018-2570-y (PMC6362582; doi:10.1186/s12859-018-2570-y)
Supplement: Supplementary file 2 — Table S2. Results of the Arabidopsis thaliana genome stepwise assembly by different assemblers using raw reads partitioned into four sets. (DOCX 13 kb) [file 12859_2018_2570_MOESM2_ESM.docx]

**Additional file 2**

**Table S2**Results of the *Arabidopsis thaliana* genome stepwise assembly by different assemblers using raw reads partitioned into four sets*

| Assembler | Assembly | Total length, Mbp | N50, bp | Number | Mean length, bp | Comparison with assembly in Table 1 using NUCMER, % | Computing time, min |
| --- | --- | --- | --- | --- | --- | --- | --- |
| Abyss | contigs | 94.82 | 2634 | 69872 | 1357 | 95,24 | 217 |
|  | scaffolds | 94,9 | 2889 | 68256 | 1390 |  |  |
| SOAPdenovo | contigs | 96,19 | 2819 | 69761 | 1378 | 73,20 | 185 |
|  | scaffolds | 97,77 | 4393 | 49114 | 1990 |  |  |
| SPAdes | contigs | 26,62 | 5098 | 18458 | 1443 | 19,77 | 248 |
|  | scaffolds | 26,65 | 5166 | 18528 | 1437 |  |  |
| CLC Assembly Cell | contigs | 113,7 | 4325 | 52576 | 2161 | 90,14 | 31 |
|  | scaffolds | 113,7 | 4450 | 50934 | 2232 |  |  |

*Minimum contig length used for assembling was 200 bp.
