# Supplementary material for: Stepwise large genome assembly approach: a case of Siberian larch (Larix sibirica Ledeb)
Source: BMC Bioinformatics. 2019 Feb 5;20(Suppl 1):37. doi: 10.1186/s12859-018-2570-y (PMC6362582; doi:10.1186/s12859-018-2570-y)
Supplement: Supplementary file 3 — Table S3. Sequencing libraries and generated sequence data used for the Larix sibirica genome assembly. (DOCX 14 kb) [file 12859_2018_2570_MOESM3_ESM.docx]

**Additional file 3**

**Table S3**Sequencing libraries and generated sequence data used for the *Larix sibirica* genome assembly

| Library^a^, tissue, insert size, bp | Raw reads | | After trimming^b^ | | Coverage, fold (X) ^c^ |
| --- | --- | --- | --- | --- | --- |
|  | Number | Total length, Gb | Number | Total length, Gb |  |
| PE, needles, 400 | 1486862072 | 297 | 1196978613 | 209 | 17.41 |
| PE, megagametophytes, 400 | 2151139430 | 430 | 1586509833 | 294 | 24.47 |
| PE, needles, 450 | 583790674 | 117 | 337089347 | 63.2 | 5.26 |
| PE, needles, 700 | 473930581 | 94.8 | 334221697 | 60.8 | 5.06 |
| MP, needles, 2-3 Kb | 1184838286 | 237 | 570430847 | 86 | 7.15 |
| MP, needles, 5-7 Kb | 805070102 | 161 | 534013725 | 90.3 | 7.5 |
| MP, needles, 8-10 Kb | 1184470381 | 237 | 610113501 | 102 | 8.45 |
| SE (unpaired after trimming) |  |  | 2235089857 | 204 | 16.99 |
| Total | 15740203052 | 1570 | 12573804983 | 1110 | 92.28 |

^a^PE – paired-end, MP – mate pair and SE – single-end libraries; ^b^Read length varied from 40 to 100 bp, 29.48% of sequence data were removed after being trimmed for poor quality, adapter sequences, bacterial contamination, etc.; ^c^assuming the length of the *L. sibirica* genome equaled 12.03 Gb [28]
