# Supplementary material for: Stepwise large genome assembly approach: a case of Siberian larch (Larix sibirica Ledeb)
Source: BMC Bioinformatics. 2019 Feb 5;20(Suppl 1):37. doi: 10.1186/s12859-018-2570-y (PMC6362582; doi:10.1186/s12859-018-2570-y)
Supplement: Supplementary file 4 — Table S4. Results of the Arabidopsis thaliana genome stepwise assembly by four different assemblers using raw reads partitioned into five sets following approach used for assembling of the Larix sibirica genome. (DOCX 14 kb) [file 12859_2018_2570_MOESM4_ESM.docx]

**Additional file 4**

**Table S4**Results of the *Arabidopsis thaliana* genome stepwise assembly by four different assemblers using raw reads partitioned into five sets following approach used for assembling of the Larix sibirica genome*

| Assembler | Assembly | Total length, Mbp | N50, bp | Number | Mean length, bp | Comparison with assembly in Table 1 using NUCMER, % | Computing time, min |
| --- | --- | --- | --- | --- | --- | --- | --- |
| Abyss | contigs | 92.58 | 3841 | 53278 | 1737 | 93.61 | 285 |
|  | scaffolds | 92.65 | 4233 | 51760 | 1790 |  |  |
| SOAPdenovo | contigs | 100.5 | 3931 | 60443 | 1501 | 76.37 | 200 |
|  | scaffolds | 101.9 | 6546 | 40160 | 2336 |  |  |
| SPAdes | contigs | 31.0 | 11232 | 12522 | 1121 | 29.03 | 291 |
|  | scaffolds | 31.1 | 11532 | 12523 | 1126 |  |  |
| CLC Assembly Cell | contigs | 115.0 | 6983 | 45293 | 2345 | 92.53 | 40 |
|  | scaffolds | 115.1 | 7045 | 44341 | 2417 |  |  |

*Minimum contig length used for assembling was 200 bp.
