# Supplementary material for: Stepwise large genome assembly approach: a case of Siberian larch (Larix sibirica Ledeb)
Source: BMC Bioinformatics. 2019 Feb 5;20(Suppl 1):37. doi: 10.1186/s12859-018-2570-y (PMC6362582; doi:10.1186/s12859-018-2570-y)
Supplement: Supplementary file 5 — Table S5. The traditional and stepwise CLC Assembly Cell genome assembly parameters for peach (Prunus persica). (DOCX 13 kb) [file 12859_2018_2570_MOESM5_ESM.docx]

**Additional file 5**

**Table S5**The traditional and stepwise CLC Assembly Cell genome assembly parameters for peach (*Prunus persica*)

| Assembly* | Method of the *de novo* assembling | Number, mln | N50, bp | Maximum length, bp | Total length, Mbp |
| --- | --- | --- | --- | --- | --- |
| Contigs | traditional | 89968 | 7692 | 103778 | 199.8 |
|  | stepwise | 89969 | 7819 | 131505 | 201.5 |
| Scaffolds | traditional | 89966 | 7692 | 103778 | 199.8 |
|  | stepwise | 89968 | 7819 | 131505 | 201.5 |

*Minimum contig length used for assembling was 200 bp.
