# Supplementary material for: Stepwise large genome assembly approach: a case of Siberian larch (Larix sibirica Ledeb)
Source: BMC Bioinformatics. 2019 Feb 5;20(Suppl 1):37. doi: 10.1186/s12859-018-2570-y (PMC6362582; doi:10.1186/s12859-018-2570-y)
Supplement: Supplementary file 6 — Table S6. The computing time taken to assemble each set and the complete Larix sibirica genome using 40 cores. (DOCX 13 kb) [file 12859_2018_2570_MOESM6_ESM.docx]

**Additional file 6**

**Table S6**The computing time taken to assemble each set and the complete *Larix sibirica* genome using 40 cores

| Assembly | Computing time, hr |
| --- | --- |
| 1 | 115.82 |
| 2 | 110.88 |
| 3 | 89.93 |
| 4 | 89.33 |
| 5 | 94.59 |
| Total | 500.55 |
| 1+2+3+MP reads | 10.20 |
| 1+2+3+4+5+MP reads | 18.28 |
